# The effect of nutrition education for cancer prevention based on health belief model on nutrition knowledge, attitude, and practice of Iranian women

**DOI:** 10.1186/s12905-022-01802-1

**Published:** 2022-06-07

**Authors:** Bahareh Sasanfar, Fatemeh Toorang, Sahar Rostami, Maryam Zarif Yeganeh, Maryam Lafzi Ghazi, Monireh Sadat Seyyedsalehi, Kazem Zendehdel

**Affiliations:** 1 Department of Nutrition, School of Public Health, Shahid Sadoughi University of Medical Sciences, Yazd, Iran; 2 Cancer Research Center, Cancer Institute of Iran, Tehran University of Medical Sciences, P.O.Box: 13145158, Tehran, Iran; 3 Department of Community Nutrition, School of Nutritional Sciences and Dietetics, Tehran University of Medical Sciences, Tehran, Iran; 4 Cancer Biology Research Center, Cancer Institute of Iran, Tehran University of Medical Sciences, Tehran, Iran; 5 Department of Community Nutrition Faculty of Nutrition Sciences and Food Technology, Shahid Beheshti University of Medical Sciences, Tehran, Iran; 6 Breast Diseases Research Center, Cancer Institute of Iran, Tehran University of Medical Sciences, Tehran, Iran; 7 Department of Exercise Physiology, Central Tehran Branch, Islamic Azad University, Tehran, Iran; 8 Department of Medical and Surgical Sciences, University of Bologna, Bologna, Italy

**Keywords:** Health Belief model, Nutrition education, Nutrition knowledge, Cancer

## Abstract

**Background:**

In recent years, nutrition has received an increasingly important role in the etiology of cancer. Thus, public education about dietary factors associated with cancer risk or prevention could be an important intervention for cancer prevention, particularly in low- and middle-income countries where the burden of cancer is increasing rapidly and the access to care is limited. The age-standardized incidence of breast cancer was 35.8 among Iranian women in 2020. We aimed to study the effect of nutrition education on the knowledge, attitude, and practice of Iranian women towards dietary factors related to cancer.

**Methods:**

In this interventional study, 229 women from public health centers were recruited and underwent three 75-min sessions of education based on the Health Belief Model (HBM). Participants were interviewed by trained interviewers using a validated and reproducible nutrition-related cancer prevention knowledge, attitude, and practice questionnaire (NUTCANKAP) questionnaire designed based on the HBM. Nutritional knowledge, attitude, and practice of participants were assessed through this questionnaire. Three 24-h dietary recalls (one weekend and two nonconsecutive weekdays) were also collected before and one month after the intervention.

**Results:**

The mean age of the participants was 45.14 years, and the mean BMI was 27.2 kg/m^2^. After the intervention, the participants had a higher intake of whole grain (*p* = 0.03) and a lower fat dairy (*p* = 0.009) and nuts (*p* = 0.04). However, the intake of high-fat dairy (*p* = 0.001) decreased after the intervention. We indicated significant differences in knowledge (*p* < 0.001) and nutritional practice scores (*p* = 0.01) after education. In addition, after the intervention, there were significant differences in the mean score of the HBM components, except for the perceived self-efficacy.

**Conclusion:**

Participation in a nutrition education program positively impacted the knowledge and nutritional practices linked to cancer prevention.

## Introduction

The burden of cancer continues to increase widely worldwide because of the population aging and increasing cancer-causing behaviors (e.g., unhealthy eating behavior, unhealthy food preparation) [1–3]. Based on the Global Cancer Observatory (GCO), which is the official cancer statistics of the International Agency for Research on Cancer (IARC), 29.5 million new cancer cases will be diagnosed worldwide in 2040 [2].

Primary prevention, including changing lifestyle and environmental interventions, has been illustrated as a key cancer control strategy for reducing this burden [4]. Previous research suggests that a combination of physical activity, having a healthy body weight, and a healthy diet could prevent one-third of cancers [5, 6]. Diet is a modifiable risk factor that can influence the risk of cancer. Several studies have investigated the relationship between dietary components, including fruit and vegetable, meat and processed meat, fiber intake, and the risk of cancer [7–9]. These studies illustrated that higher consumption of red and processed meat might increase breast cancer risk. However, adherence to a prudent dietary pattern containing high fruits, vegetables, and fibers might decrease breast cancer risk [10]. Furthermore, greater levels of nutrition knowledge have been linked to higher health literacy, better management of chronic diseases, and lower health costs [11]. Changin of attitude and practice has increasingly been used in nutrition education to improve intervention efficacy. Nutrition education programs could help to increase nutrition knowledge and improve dietary behaviors which may reduce the incidence of many chronic diseases including, cancer, diabetes, and cardiovascular disease [12]. Sullivan et al. illustrated that a nutrition education program strengthened nutrition-related cancer prevention attitudes among low-income African American women [13]. Another study assessed the effects of education on dietary behavior and showed that education plans based on HBM could change nutritional beliefs and behaviors for colorectal cancer prevention [14]. Regarding dietary intake behavior, an ecologic study and a meta-analysis of prospective studies found a positive relation between habitual salt intake and risk of gastric cancer [15, 16].

The Health Belief Model (HBM) is one of the most recommended models in the field of nutrition education programs [17]. This model describes the risks of unhealthy behaviour and the related and understands their susceptibility to adverse outcomes of their feelings and can be used as motivation to reduce risks [18]. This model includes five components: perceived susceptibility (i.e., the level in which a person knows his sensitiveness about a disease), perceived severity (i.e., the perceptions of the person about the severity of the disease), perceived benefits (i.e., the person’s understanding about the advantages of the preventive behavior), perceived barriers (i.e., each healthy behavior and practice may encounter some barriers and problems), and performance guides (i.e., stimulations, which facilitate decision-making) [19–21]. Some studies have stated the benefits of using this model in different health education programs [22, 23]. The use of the HBM is appropriate for myriad preventive health behaviors among both men and women; mainly, it has been a directive to researchers studying women’s health issues [24]. In addition, nutrition researchers have frequently applied the HBM in cancer prevention education.

To our knowledge, there are limited studies about the effect of nutrition education on knowledge, attitude and practice for cancer prevention in Middle-Eastern countries. Therefore, we designed a prospective study in Iran to: (1) Assess the effect of nutrition education based on the HBM on the nutritional knowledge, attitude, and practice (KAP); (2) Recognize perceived barriers to the adherence of eating behaviors related to cancer prevention; (3) Change in nutritional behavior including food choice and methods of food processing that are associated with cancer prevention.

## Subjects and methods

There were 229 participants who were visiting public health centers. We invited the women to participate in the study through flyers, posters, and introduction of this study on social media. We conducted an interventional study and used a one-group pretest–posttest design, through convenience sampling in 2017–2018, to evaluate the impact of nutrition education on the knowledge, attitude, and practice of women referring to the public health centers of Tehran University of Medical Science, located in Iran. The participating women were interviewed by trained interviewers using a validated and reproducible the nutrition-related cancer prevention knowledge, attitude and practice 36 questionnaire (NUTCANKAP) [25]. A 24-h dietary recall was conducted by phone on three different days, including two non-consecutive weekdays and a weekend before and one month after the intervention for all participants. The NUTCANKAP questionnaire was designed based on the HBM and consisted of three sections: A. knowledge (10 questions), B. attitude (27 questions; including 11 questions on perceived susceptibility, four questions on perceived severity, four questions on perceived benefits, four questions on perceived self-efficacy, and four questions on perceived barriers), and C. practice (16 questions). Correct answers in the knowledge section were given a score of 1. Incorrect answers, don’t know-answers, and blanks were assigned a score of zero. The total raw scores of knowledge ranged from 0 to 10. The attitude section was evaluated by a Likert scale ranging from 1 as least desirable to 5 as most desirable, or vice versa. In the items related to the practice domain, correct food choices received a score of 1, and incorrect or blank responses were regarded as zero (Table 1). We calculated intakes of energy and all consumed foods through three recalls and then converted them to grams by a program made by the authors in Microsoft Access. The nutrient composition of consumed foods was determined based on the USDA food composition database modified for Iranian foods. Participants who met the inclusion criteria were literate women aged 19–70 who had an available phone number for follow-up. Exclusion criteria included not being interested in continuing the study or being on dietary restrictions. Written informed consent was obtained from each participant.

**Table 1:** Summary of item content and scoring of the NUTCANKAP questionnaire

Domain/constituent	No. of item	Example question	Scoring
Knowledge of cancer risk factors	10	Less intake of fried food can help to cancer prevention Higher intake of salt was associated with a risk of cancer	1 = Correct answer 0 = Incorrect or don't know-answer
Attitude towards cancer prevention Perceived susceptibility Perceived severity Perceived benefits Perceived self-efficacy Perceived barriers	27 11 4 4 4 4	Method and time of food storage is important in cancer prevention Treatment cost are high for cancer Sufficient vitamin D status may helps to cancer prevention I can consumption moldy food after removing the mold from suface of food and heating it Flatulence from beans is a barrier to consuming them	5 = Strong agree 4 = Agree 3 = Don’t know 2 = Disagree 1 = Strongly disagree (or vice versa)
Practice for cancer prevention	16	I choose fruit juice rather than cola for beverage I choose boiling process rather than frying for food preparation	1 = Correct food choices 0 = Incorrect or blank responses

After filling out the questionnaire, the educational program was performed in three 75-min sessions. Subjects were also given a book on cancer prevention through healthy nutrition. The educational program was designed based on the components of the HBM and pretest results was conducted through live lectures, collaborative question-answering methods, group discussions, and visual education materials such as slide shows. In the first session, the health educator informed them about cancer, potential risk factors, obesity and cancer, and healthy and unhealthy foods concerning cancer, through presentation slides. In the second session, the health educators held group discussions about the topics of previous session, types of cooking methods, and cooking dish. The educators also tried to promote attitude toward behavior in the participants. Information on food nutrition labels and the five food groups based on the food pyramid for reforming nutritional behavior was discussed in the third session [26]. In addition, a woman who had lost her first- or second-degree relative (s) due to cancer was invited to talk about the severity of the consequences of the disease. All procedures involving human subjects/patients were approved by the Research Ethics Committee of Tehran University of Medical Science (code: 28,614).

Finally, data were analyzed by STATA version 14 (State Corp., College Station, TX). The chi-square test and t-test were used for qualitative and continuous variables, respectively. Multivariable logistic regression was used to estimate the association between KAP scores and age, educational and socioeconomic status. Age and socioeconomic were controlled as covariates.

## Results

The mean age of the participants was 45.14 years (standard deviation = 10.16, range from 20 to 70), and the mean BMI was 27.2 kg/m^2^. The majority of subjects were overweight (37.33%) and had a diploma (65.28%). Table 2 shows the association between KAP scores and age, educational, and socioeconomic status before the intervention. Women with university education had higher knowledge (*p* = 0.01) and nutritional practice (*p* = 0.04) scores than those with primary education. However, no significant differences were observed across age and socioeconomic groups. After the intervention, women reduced intake of carbohydrate (*p* = 0.008), total protein (*p* = 0.03), animal protein (*p* = 0.05), vegetable fat (*p* = 0.01), saturated fatty acid (*p* = 0.0002), monounsaturated fatty acid (*p* = 0.04), Cobalamin (*p* = 0.01), Iron (*p* = 0.01), and Selenium (p = 0.006) (Table 3). In addition, after the intervention subjects had higher intake of whole grain (*p* = 0.03), low fat dairy (*p* = 0.009), and nuts (*p* = 0.04). However, the intake of high-fat dairy (*p* = 0.001) decreased (Table 4) after education. Comparisons of the scores of knowledge, attitude (perceived susceptibility, perceived severity, perceived benefits, perceived barriers, and perceived self-efficacy) and nutritional practice, before and after the education are presented in Table 5. We indicated significant differences in knowledge (*p* < 0.001) and nutritional practice scores (*p* < 0.001) following education. Moreover, after the intervention, there were significant differences in attitude (*p* < 0.001). Therefore, the mean score of knowledge, attitude and nutritional practice significantly increased after the intervention. We found a significant association between the improvements of attitude score after the intervention (P_trend_ = 0.04) and the level of education (Table 6). The association was also significant for the specific question of knowledge about BMI and the level of education (OR = 6.27; 95% CI = 1.72–22.7, P_trend_ = 0.001) and socioeconomic status (OR = 0.39; 95% CI = 0.18–0.83, P_trend =_ 0.01). In addition, we found a significant association between sing food labels and the level of education (OR = 6.07; 95% CI = 1.71–21.5, P_trend_ = 0.006).

**Table 2:** The association of studied KAP scores before the intervention between age, educational and socioeconomic groups

	Knowledge	Attitude	Practice
Age^1^	OR (CI)	OR (CI)	OR (CI)
20–41	Reference	Reference	Reference
42–50	0.56 (0.27–1.16)	1.42 (0.69–2.92)	1.07 (0.52–2.18)
51–69	0.86 (0.41–1.81)	1.46 (0.70–3.05)	1.84 (0.87–3.90)
^†^*P* for trend	0.48	0.33	0.13
Educational status^2^			
Primary	Reference	Reference	Reference
Diploma	3.98 (1.21–13.10)	1.50 (0.52–4.30)	2.49 (0.85–7.29)
University	5.49 (1.47–20.39)	1.14 (0.33–3.35)	3.67 (1.09–12.37)
^†^*P* for trend	**0.01**	0.79	**0.04**
Socioeconomic status^2^			
Low	Reference	Reference	Reference
Medium	1.84 (0.87–3.90)	1.33 (0.63–2.81)	1.22 (0.58–2.56)
High	1.07 (0.51–2.21)	0.80 (0.39–1.66)	1.11 (0.53–2.31)
^†^*P* for trend	0.76	0.52	0.73

Significant* P* value (< 0.05) was bolded

^1^ adjusted for Socioeconomic status

^2^ adjusted for age

†Obtained from logistic regression. OR has been computed by considering subjects over medium scores as one and lower medium scores as zero

**Table 3:** Comparison of macro- and micronutrient intake before and after the intervention

	Before intervention	After intervention	*p*-value
Energy (kcal)	2200.5 ± 62.0	2117.7 ± 60.0	0.11
Carbohydrate (g/d)	295.29 ± 52.59	282.72 ± 46.54	**0.008**
Total protein (g/d)	66.37 ± 15.52	62.80 ± 21.87	**0.03**
Animal protein (g/d)	35.27 ± 24.87	31.35 ± 18.13	**0.05**
Vegetable protein (g/d)	37.98 ± 8.60	37.85 ± 8.80	0.44
Total fat (g/d)	60.22 ± 21.04	59.22 ± 21.14	0.32
Animal fat (g/d)	27.09 ± 14.03	25.33 ± 14.99	0.13
Vegetable fat (g/d)	40.09 ± 21.97	35.37 ± 19.80	0.01
Saturated fatty acid (g/d)	20.38 ± 9.06	17.15 ± 10.34	**0.0002**
Monounsaturated fatty acid (g/d)	19.95 ± 8.03	18.56 ± 7.63	**0.04**
Polyunsaturated fatty acid (g/d)	16.62 ± 12.27	16.61 ± 11.10	0.49
Cholesterol (mg)	160.00 ± 116.38	156.07 ± 86.83	0.36
Fiber (g/d)	20.26 ± 10.88	19.90 ± 7.17	0.32
Vitamin C	159.84 ± 131.67	154.82 ± 100.66	0.32
Vitamin A (µg)	855.33 ± 792.55	839.79 ± 589.52	0.40
Thiamin (mg)	1.60 ± 0.35	1.55 ± 0.36	0.10
Riboflavin (mg)	1.35 ± 0.49	1.30 ± 0.48	0.09
Niacin (mg)	17.62 ± 27.32	15.84 ± 33.83	0.30
Pyridoxin (mg)	1.43 ± 0.49	1.36 ± 0.46	0.06
Cobalamin (µg)	1.22 ± 2.59	0.21 ± 3.66	**0.001**
Folate (µg)	279.78 ± 10.63	282.61 ± 11.15	0.58
Iron (mg)	20.42 ± 9.38	18.78 ± 7.69	**0.01**
Calcium (mg)	899.70 ± 342.19	865.14 ± 347.28	0.09
Zinc (mg)	7.59 ± 3.02	7.27 ± 1.73	0.11
Selenium (µg)	80.48 ± 3.10	77.67 ± 3.02	**0.006**

Significant* P* value (< 0.05) was bolded

Values are mean (SD)

**Table 4:** Comparison of food group intake before and after the intervention

Food group	Before intervention	After intervention	*p*-value
Wholegrain	24.92 ± 64.49	37.17 ± 59.27	**0.03**
Refined grain	217.57 ± 114.63	231.02 ± 131.52	0.14
Low-fat dairy	136.27 ± 152.25	166.68 ± 146.31	**0.009**
Medium fat dairy	27.78 ± 80.76	30.32 ± 71.53	0.37
High-fat dairy	135.37 ± 191.41	83.37 ± 150.66	**0.001**
Meat	70.117 ± 108.27	65.78 ± 73.18	0.33
Processed meat	10.77 ± 39.30	7.91 ± 31.82	0.22
Fish	8.05 ± 21.57	10.49 ± 29.40	0.16
Vegetables	318.32 ± 189.65	313.12 ± 229.15	0.40
Fruits	522.79 ± 297.39	560.79 ± 275.61	0.07
Fruit juice	34.27 ± 86.58	30.90 ± 67.18	0.33
Beans	36.06 ± 50.87	32.40 ± 54.27	0.27
Sweat and desserts	15.65 ± 54.38	16.67 ± 42.22	0.42
Hydrogenated fat	4.14 ± 17.39	4.33 ± 9.54	0.45
Animal fat	3.78 ± 8.17	4.67 ± 11.70	0.18
Olive and other vegetable oil	5.20 ± 17.72	5.31 ± 12.37	0.47
Egg	9.82 ± 27.61	9.09 ± 27.08	0.40
Nuts	4.27 ± 23.44	8.95 ± 28.71	**0.04**

Significant* P* value (< 0.05) was bolded

Values are mean (SD)

**Table 5:** Comparison of studied KAP scores before and after the intervention

Variable	Before intervention	After intervention		*p*-value
	Mean (± SD)	Mean (± SD)	Differences(± SD)	
Knowledge†	33.83 (10.05)	41.12 (7.32)	8.14 (9.29)	< 0.001
Attitude†	62.22 (18.45)	71.82 (19.23)	9.59 (20.9)	< 0.001
Perceived susceptibility	44.71 (5.13)	45.96 (4.60)	1.24 (3.45)	0.001
Perceived severity	65.54 (21.62)	77.12 (16.94)	11.57 (23.13)	< 0.001
Perceived benefits	59.61 (16.72)	65.23 (13.52)	5.62 (19.33)	0.0002
Perceived barriers	77.27 (40.05)	89.93 (29.91)	12.66 (42.99)	0.0002
Perceived self-efficacy	78.95 (17.23)	84.40 (18.61)	5.44 (21.74)	0.001
Practice†	69.56 (15.92)	79.19 (12.83)	9.62 (15.14)	< 0.001

SD standard deviation

*P*-values were determined by the T-test

†The values are shown in the scale of 0–100

**Table 6:** The association of differences of KAP scores between age, educational and socioeconomic groups†

	Differences in knowledge score		Differences in attitude score	Differences in practice score		
	Overall	Q3^⁕^	Overall	Overall	Q14^⁕⁕^	Q17^⁕⁕⁕^
Age^1^	OR (CI)	OR (CI)	OR (CI)	OR (CI)	OR (CI)	OR (CI)
20–41	Reference	Reference	Reference	Reference	Reference	Reference
42–50	0.87 (0.42–1.80)	0.97 (0.46–2.01)	0.97 (0.46–2.04)	1.13 (0.55–2.31)	0.79 (0.38–1.63)	0.96 (0.41–2.23)
51–69	1.29 (0.60–2.77)	1.59 (0.74–3.39)	0.94 (0.44–1.99)	0.84 (0.41–1.74)	1.40 (0.65–3.03)	1.06 (0.42–2.65)
^†^*P* for trend	0.54	0.25	0.87	0.69	0.46	0.91
Educational status^2^						
Primary	Reference	Reference	Reference	Reference	Reference	Reference
Diploma	1.27 (0.44–3.60)	1.21 (0.42–3.46)	1.09 (0.38–3.10)	1.23 (0.43–3.48)	3.15 (1.06–9.29)	0.76 (0.23–2.48)
University	2.17 (0.66–7.11)	6.27 (1.72–22.7)	2.75 (0.80–9.46)	1.72 (0.53–5.55)	6.07 (1.71–21.57)	0.52 (0.12–2.09)
^†^*P* for trend	0.13	**0.001**	**0.04**	0.29	**0.006**	0.36
Socioeconomic status^2^						
Low	Reference	Reference	Reference	Reference	Reference	Reference
Medium	0.89 (0.41–1.89)	0.71 (0.33–1.52)	0.96 (0.45–2.06)	1.17 (0.56–2.44)	0.69 (0.32–1.49)	1.19 (0.48–2.90)
High	0.60 (0.28–1.28)	0.39 (0.18–0.83)	0.99 (0.46–2.11)	0.92 (0.44–1.90)	0.57 (0.26–1.22)	1.84 (0.77–4.42)
^†^*P* for trend	0.21	**0.01**	0.98	0.82	0.15	0.16

Significant* P* value (< 0.05) was bolded

^1^adjusted for socioeconomic

^2^adjusted for age

^⁕^ Q3 = What is the normal range of BMI for adults?

^⁕⁕^ Q14 = Do you use food labels when choosing food?

^⁕⁕⁕^ Q17 = Do you use high-fat dairy?

^†^Obtained from logistic regression. OR has been computed by considering subjects over medium scores as one and lower medium scores as zero

Comparison of the scores obtained from question 17 of nutritional practice before and after the intervention is shown as a sample in Table 7.

**Table 7:** Comparison of question 17 scores of practice before and after the intervention (question 17: which of the following do you do?)

	Before	After	P*
The overall score of practice question 17	0.24 ± 0.21	0.42 ± 0.36	< 0.001
I put my hands and feet for a few minutes against the sun to make vitamin D	0.26 ± 0.44	0.37 ± 0.48	0.003
I check my iron and folic acid levels under the guidance of a nutrition consultant	0.36 ± 0.48	0.64 ± 0.47	0.0000
I will reduce the consumption of simple sugar like sweets	0.74 ± 0.43	0.92 ± 0.26	0.0000
I don’t use soda even zero types	0.53 ± 0.50	0.70 ± 0.45	0.0002
I check my weight every week	0.41 ± 0.49	0.53 ± 0.50	0.001
I use low-fat dairy instead of high-fat dairy	0.70 ± 0.45	0.77 ± 0.41	0.04

^*^ Obtained from independent student's t-test

## Discussion

This study is the first study that used the HBM in nutrition education for cancer prevention in Iran. We concluded that the application of the HBM in nutrition education for cancer prevention could result in promoting the level of knowledge, attitude, and nutritional practice among Iranian women. Our results showed a low level of knowledge about cancer causes, protective nutrients and those lowering the risk of cancer, healthy cooking methods, food guide pyramid, and healthy cooking dish before the intervention. The score of knowledge was higher among women with a university education than other groups before the intervention (*p* < 0.01). Knowledge of participants about normal BMI range doubled after education (0.14 vs. 0.28). However, the mean score of knowledge significantly increased after the intervention. There was a non-significant increasing trend in the difference in knowledge score between age and education. However, the before-after differences in knowledge and practice among participants with a high socioeconomic status were lower than those at the higher socioeconomic level. This may suggest that higher economic levels do not necessarily reflect greater awareness. Multiple levels of influence affect an individual's food choice. Biological and cultural influences such as taste, sex, and age may have significant effects on food consumption [27, 28]. This suggests that food consumption is not necessarily associated with the cost [29]. on the other hand, families have to pay rent, clothes, and transport in addition to buying food. Therefore, only a tiny part of their income is allocated to food.

Ahn et al. [30] showed that nutritional education positively impacted dietary habits and nutritional knowledge in older adults. A group of researchers in the USA conducted a program to teach children about cancer and cancer control behaviors and found it successful in promoting the knowledge about cancer risk factors, forming a positive attitude towards cancer risk factors, and increasing cancer control behaviors among students [31]. It should be kept in mind that nutrition literacy enables people to use written information related to health. Therefore, increasing nutrition knowledge has a protective effect against diseases. A systematic review illustrated that the majority of studies reported a significant association between nutrition knowledge and dietary intake [11].

We used the HBM to increase the impact of nutrition education. The HBM would seem to be used widely for communication research [32], and has been suggested [33] and approved [34] as a model for nutrition education. In our study, the score of the HBM constructs, including perceived susceptibility, perceived severity, perceived benefits, perceived barriers, and perceived self-efficacy increased following a nutrition education program. Additionally, in this study, the score of perceived barriers increased after the intervention. This implies that women recognized the barriers and would try to resolve them. Similarly, an interventional study on gastric cancer among 84 Iranian housewives showed that the intervention group based on the HBM model showed significantly higher scores after the education [35]. Attitude scores showed a decreasing trend between age, educational, and socioeconomic status before the intervention. However, the difference in attitude scores had an increasing trend after the intervention unless the socioeconomic status. Meanwhile, an interventional study on 157 African American women on nutrition-related cancer prevention showed that attitudes improved after a nutrition education program [13].

This study illustrated that nutrition education program based on the HBM has a positive effect on food choices among women. We assessed this change through a questionnaire and three recalls and found that the score of nutritional practice increased after the intervention.

The question score on food labeling usage increased by 82%, and the question score of high-fat dairy usage increased by 33% after the education. Therefore, participants had a better food choice following nutrition education. An increase in whole grain, low-fat dairy, and nuts was also found after the intervention. These food groups compose of nutrients protecting against cancer [36]. In addition, we found a decrease in carbohydrate, total protein, animal protein, vegetable fat, saturated fatty acid, monounsaturated fatty acid, Cobalamin, Iron, Selenium, and high-fat dairy. Studies illustrated that carbohydrate intake is positively associated with cancer via insulin and the related hormone, IGF-1 [37–39]. We found significant associations for the specific question of knowledge on BMI and level of education (OR = 6.44) and socioeconomic status (OR = 0.39). Similarly, differences in knowledge, attitude and nutritional practice scores between socioeconomic groups showed a decreasing trend after the intervention. Jing Wu et al. [40] suggested that a higher intake of total red meat, fresh red meat, processed meat, and high-fat dairy may be risk factors for breast cancer. Regarding fatty acids, various studies have demonstrated that polyunsaturated fatty acids possess a therapeutic role against certain types of cancer [41]. In contrast, the intake of saturated fatty acids has been linked to cancer [42]. A study evaluated medical students' knowledge about the association between dietary factors and the risk of cancer and indicated that diet-disease knowledge was higher among those who had a higher dietary fiber intake [43]. In our study, increasing in knowledge score was seen toward vegetables and fruits consumption, but it was not achieved in nutritional practice scores. The barriers to low fruit intake in participants were determined through the questionnaire. Limited budget was mentioned by 13.4% of participants as the main barrier to fruit consumption. Around 10% of the participating women believed that preparing fruits is time-consuming which could be a barrier to fruit intake, and 2.59% limited their fruit consumption due to digestive problems. A small part of our study sample (1.04%) cut fruit intake because they believe fruits are contaminated with toxins. In addition, participants reported a lack of vegetable consumption due to difficulty in preparation (18.75%), cost (2.07%), lack of irrigation with safe water (8.29%), or digestive problems (8.29%).

A study conducted in Northwest of Iran illustrated that the food habits of East-Azerbaijan people in the last two decades increase the risk of gastric cancer and suggested performing nutrition education for a healthy diet [44]. In the Golestan cohort study, the incidence of esophageal cancer was associated with nutrient intake and dietary behaviors such as polycyclic aromatic hydrocarbons and drinking hot tea [45]. Other Iranian studies indicated that nutrition-related attitudes were positively correlated with the dietary practices of breast cancer prevention [46]. Since Iranian unique dietary habits are modifiable by education and with regards to the burden of high health system costs of cancer imposed on patients and the government, the application of education programs would be cost-effective.

This study was limited to the intervention group. Our study was done before and one month after the intervention, which only showed the short-term effects of the intervention. The study population was limited to females. Women have a critical role in food choices and nutrition education of children in the family. Due to strong linkage between maternal education and children’s health, we conducted this study among women. However, the results of this study cannot be generalized to men, and additional research among Iranian men is needed. Some confounding variables such as personality characteristics, mental health, and media might have affected the outcome, which was not assessed. According to the differences in scores between different age groups, a different educational approach may have to be applied to each age group. The strengths of the current study include large sample size, recruiting participants from various areas of Tehran, and the use of visual education materials. We also used a validated instrument to measure educational intervention and assessed food practice by collecting dietary recalls, which have not been done by many studies [25].

In conclusion, this study showed that a nutrition education program based on the HBM had a positive impact on the knowledge and nutritional practice of Iranian women. Considering the cost-effectiveness of educational programs compared to treatment services, applying health education programs can highly promote public health.

## Data Availability

Data is a available on reasonable request by corresponding author (Dr. Kazem Zendehdel).
